# Direct observation of dislocation plasticity in high-Mn lightweight steel by *in-situ* TEM

**DOI:** 10.1038/s41598-019-51586-y

**Published:** 2019-10-23

**Authors:** Sung-Dae Kim, Jun Young Park, Seong-Jun Park, Jae hoon Jang, Joonoh Moon, Heon-Young Ha, Chang-Hoon Lee, Jun-Yun Kang, Jong-Ho Shin, Tae-Ho Lee

**Affiliations:** 1 Advanced Metals Division, Korea Institute of Materials Science, 797 Changwondaero, Changwon, 51508 Republic of Korea; 2 Materials and Manufacturing Technology Development Center, Corporate Research and Development Institute, Doosan Heavy Industries and Construction Co. LTD., 22 Doosanvolvo-ro, Changwon, Gyeongnam, 51711 Republic of Korea

**Keywords:** Engineering, Materials science

## Abstract

To gain the fundamental understanding of deformation mechanisms in an aluminum-containing austenitic high-Mn steel (Fe-32Mn-8.9Al-0.78 C (wt.%)), *in-situ* straining transmission electron microscopy (TEM) analysis is conducted. The *in-situ* observation during the deformation demonstrates that the plastic deformation is accommodated by the pronounced planar dislocation gliding followed by the formation of slip bands (SBs) and highly dense dislocation walls (HDDWs). Experimental evidences of the glide plane softening can be obtained from the interaction between the gliding perfect dislocations and the L’1_2_ ordered precipitates in the austenite matrix. Furthermore, the observation of the localized cross-slip of dislocations at the slip band intersections enables to understand why slip bands are extensively developed without mutual obstructions between the slip bands. The enhanced strain hardening rate of the aluminum-containing austenitic high-Mn steels can be attributed to the pronounced planar dislocation glides followed by formation of extensive slip band which prevent premature failure by suppressing strain localization.

## Introduction

High-Mn steels (HMnS) gather much attention due to their superior mechanical properties including the combination of high strength and enhanced ductility^1,2^. These outstanding features of the alloys are enabled by maintaining high strain hardening rate up to relatively higher strain level, whereas common metallic alloys show a monotonic decrease of strain hardening with ongoing strain^3^. The characteristic strain hardening phenomena are generally attributed to their unique hardening mechanisms such as transformation-induced plasticity (TRIP)^4,5^, twinning-induced plasticity (TWIP)^2,6^, shear-band-induced plasticity (SIP)^7^, microband-induced plasticity (MBIP)^8,9^, slip band refinement-induced plasticity (SRIP)^10^ and dynamic slip band refinement (DSBR)^11,12^. It is generally understood that the activation of the dominant hardening mechanism is mainly determined by the stacking fault energy (SFE) of austenite matrix which can be adjusted by alloy design utilizing additions of alloying elements (Mn, C, Al, etc.)^13–16^. Following the conventional wisdom, the deformation behaviors of low-SFE (<20 mJ/m^2^) and medium-SFE (20–40 mJ/m^2^) alloys have been characterized by the TRIP and TWIP mechanisms, respectively^2,16–19^.

Aluminum-containing austenitic high-Mn steels (Fe-Mn-Al-C steels) have been widely revisited as one of the most promising subgroups of the high-Mn steels because of their attractive mechanical properties as well as low specific weight^7,8,11,18–20^. Addition of 1 wt.% Al reduces 1.3~1.5% of specific weight, rendering the alloys to be so-called ‘lightweight steels’ and thus to be an encouraging candidate for automotive applications^7,18,19^. Because an addition of Al increases the SFE, the lightweight high-Mn steels have SFEs in the range between 80 mJ/m^2^ and 120 mJ/m^2^ ^18,19^. Considering the high level of SFE, it is expected that both of TRIP and TWIP are not activated; instead plastic deformation is materialized mainly by non-dissociated perfect dislocations gliding^8,11,21^. Also, in the high-SFE fcc materials, screw components of gliding dislocations are easy to change their glide planes via cross-slip leading to form the peculiar dislocation substructure known as ‘wavy glide’^22^. However, in the Fe-Mn-Al-C steels, planar dislocation glide is frequently observed even with high SFE^7,8,10,11^. The reason of such a unique deformed structure of the alloys has been controversially discussed. The majority of previous studies attributes the origin of the peculiar deformation microstructure to the ordered phase in the alloys^7,8,10,11,18,19,23^, that is, the ordered phases dispersed in the austenite matrix hinder dislocation movement. On the other hand, if an ordered phase is sheared by some preceding dislocations, then the succeeding dislocations will be easy to glide on the same slip plane (“glide plane softening”).

Although, many researchers agreed the validity of the “glide plane softening” effect, disagreements remain about the interpretations of the strain hardening process in the alloys. Frommeyer *et al*.^7^ suggested that the finely dispersed κ-carbides (Fe-Mn-Al-C TRIPLEX steels) lead to the planar glide of dislocations accompanied by the homogeneous shear band formation. Even they confirmed the pronounced planar glide in the Fe-Mn-Al-C steels, detailed microscopic explanations regarding the enhanced strain hardening rate was not clearly provided. According to the MBIP mechanism^8,9,24^, the extended elongations are originated from strain accommodation by forming the Tayler lattice (TL) at low strains. Upon further deformation, the additional strains are accommodated by rotation of TL followed by formation of domain boundaries (DBs) and microbands (MBs). Even the MBIP supports the pronounced planar glide in the Fe-Mn-Al-C steels, it emphasizes the grain subdivision and explains the origin of the enhanced strain hardening rate in terms of the Hall-Petch effect. The slip band mediated plasticity mechanisms for the Fe-Mn-Al-C steels including the DSBR^11^ and the SRIP^10^ have been widely appreciated in recent times. They validate microscopic evidences of the planar dislocation glide, and suggest that the slip band formation and refinement during the plastic deformation are the main source for the enhanced strain hardening rate of the Fe-Mn-Al-C steels.

Despite lots of investigations, diverse *post-mortem* analyses of the band-like deformed microstructure (shear band^7^, micro-band^8,24^, slip band^10,11^) in the the Fe-Mn-Al-C steels have been causing a controversy about the underlying deformation mechanisms. Even though all of the suggested mechanisms agree the planar dislocation glide during plastic deformation originated from the existence of the fine ordered phase in the austenite matrix, each of the mechanisms interprets the resultant dislocation substructure after the deformation in different ways. For example, the MBIP^8,24^ explains that the band-like microstructure is the domain boundary which is a dislocation wall consisting of geometrically necessary dislocations to accommodate the misfit orientation between the Taylor lattice domains, and emphasizes the dynamic Hall-Petch effect from the microstructural refinement by formation of the multiple domain walls. However, SRIP^10^ and DSBR^11^ interpret the band-like structure as the slip band composed of pile-up dislocations, and the long-range interaction between the pile-up dislocations is highlighted in the mechanisms. We have thought that this discrepancy might arise because the *post-mortem* investigation of the resultant microstructure cannot give any information about the intermediate status during the plastic deformation. Therefore, we have tried to conduct *in-situ* straining TEM experiments to observe the dynamic evolution of the band-like structures in the alloys. The present study aims to elucidate the deformation mechanism of the Al-added austenitic high-Mn steels by means of transmission electron microscopy (TEM). Especially, we conduct *in-situ* mechanical straining TEM experiments to show the dynamic evolution of the deformed microstructure including the dislocations gliding behavior. We demonstrate the nature of the pronounced planar glide of the dislocations and of the dislocation interaction at the slip band intersection. In comparison with the previous *post-mortem* analysis, our *in-situ* TEM observation provides a clue towards in-depth understanding of plasticity underlying the high strain hardening capacity of the alloys.

## Results

**Figure 1:**
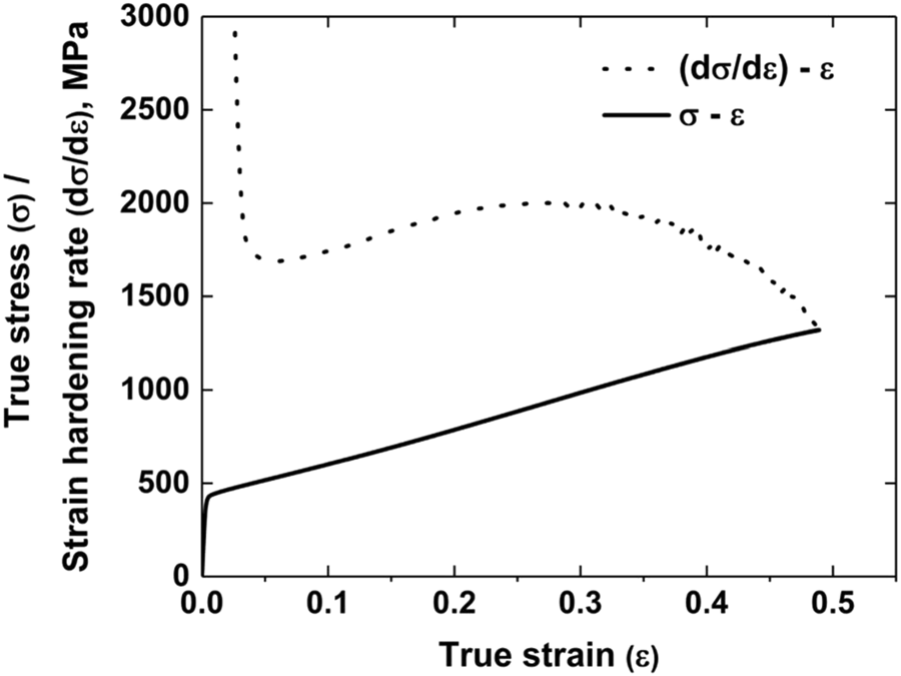
True stress-strain curve (solid line) and strain hardening rate curve (dashed line) of the alloy in the as-quenched state. The ‘hump’ in the strain hardening rate reveals a dominant hardening mechanism is activated with ongoing strain.

Figure 1 shows true stress-strain curve (solid line) and strain hardening rate (dashed line) at room temperature. The material exhibits yield strength of 425 MPa, maximum strength of 1319 MPa and uniform elongation of 0.49. These excellent mechanical properties are comparable to those of the Fe-Mn-Al-C steels with similar composition^11,18,19^. After the yield point, the strain hardening rate is gradually increased to the maximum value of 2000 MPa and decreased until fracture. The ‘hump’ in the strain hardening rate curve implies a dominant hardening mechanism is activated with ongoing strain. However, any distinct transition in the hardening rate as in the case of Fe-30.5Mn-2.1Al-1.2 C alloy^25^ is not observed in the current alloy. To find out the dominant hardening mechanism, we conducted *post-mortem* TEM analyses to identify the precipitates in the ordered austenite matrix and *in-situ* straining TEM experiments to visualize the time-resolved dynamic microstructure with ongoing strain.

**Figure 2:**
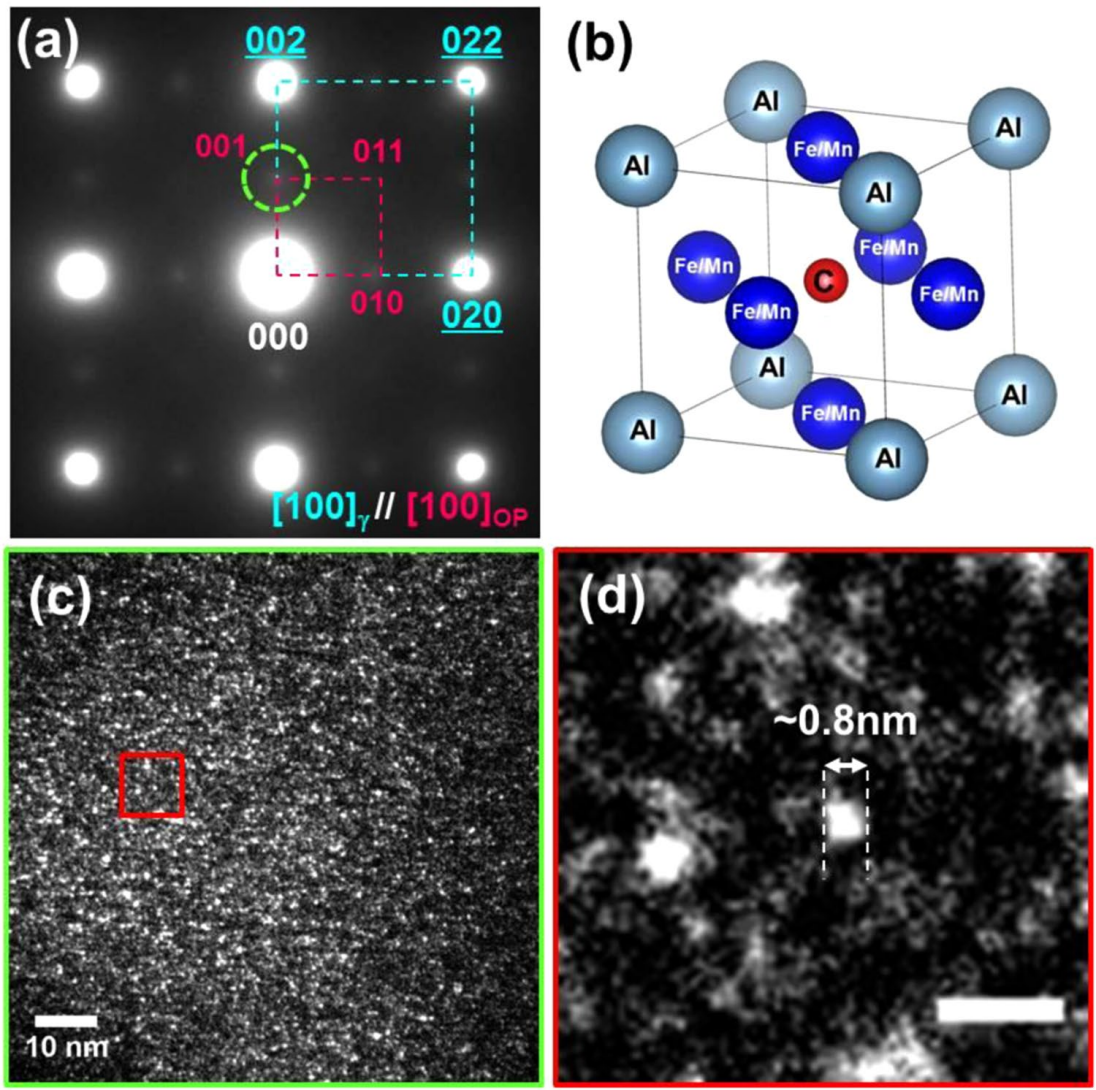
(**a**) Selected area diffraction pattern (SADP) of the as-quenched alloy along with [001]-zone axis. The faint superlattice reflections show presence of the ordered phase precipitates. **(b)** Atomic structure of the κ-carbide (E2_1_ structure). **(c)** Dark-field (DF) TEM micrograph taken by using the (001) reflection of the ordered phase (green dotted circle in (**a**)). Fine precipitates are dispersed in the matrix. **(d)** Magnified DF image extracted from the region with red square in (**c**). Length of the scale bar is 2 nm.

**Figure 3:**
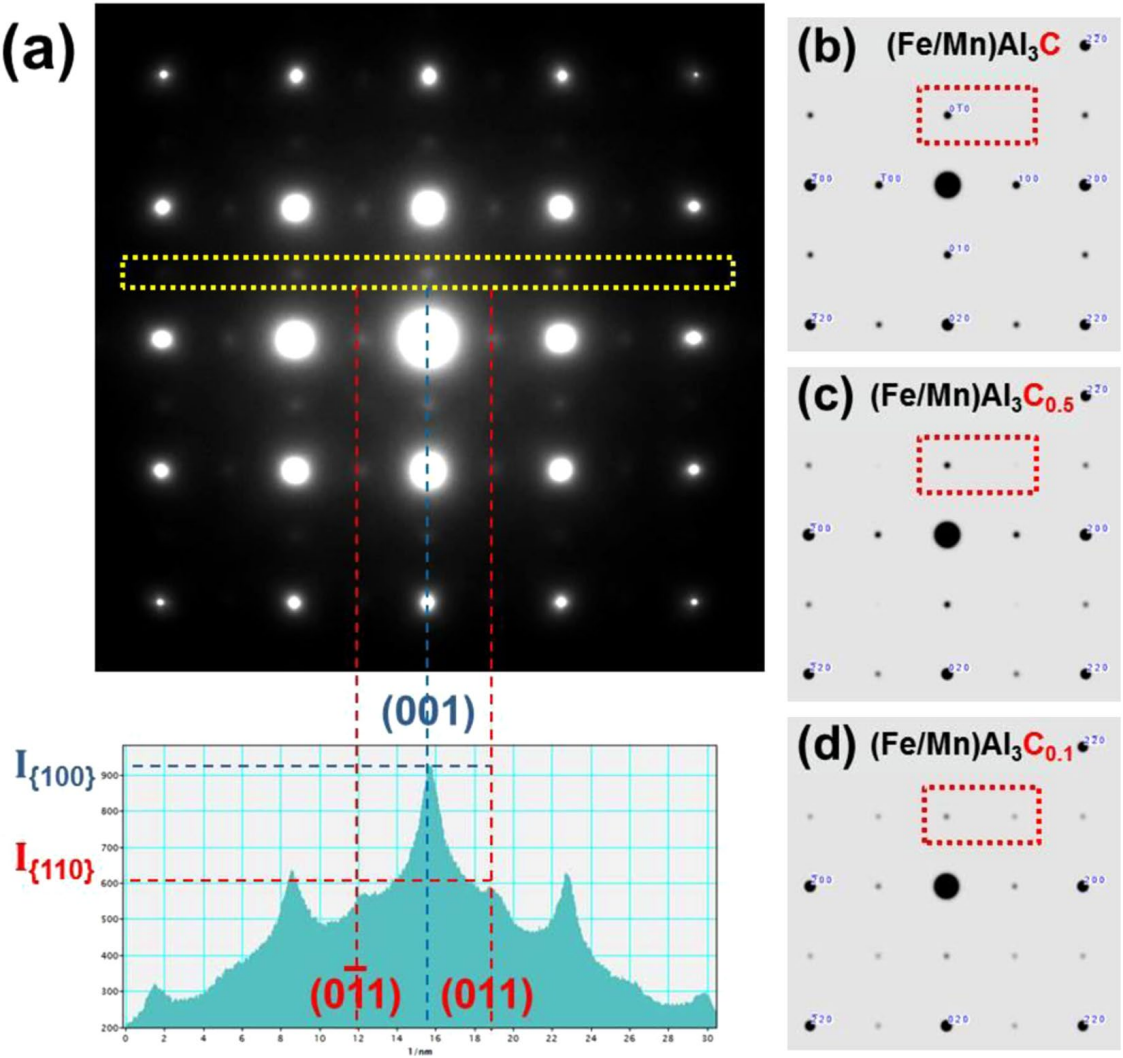
(**a**) Selected area diffraction pattern (SADP) of the as-quenched alloy along with [001]-zone axis (Upper). Intensity profile from the yellow dotted rectangular box in the SADP (Lower). (**b**–**d**) simulated SADPs of the L’1_2_ phase with decreasing C contents of 20, 10 and 2 at.%, respectively.

A TEM selected-area electron diffraction pattern (SADP) of the as-quenched alloy is shown in Fig. 2a. The pattern exhibits the reflections of austenite matrix (marked with cyan dotted square) and faint superlattice reflections of ordered phase (magenta dotted square) along the [001]-zone axis. Based on the superlattice reflections from precipitates, it is confirmed that the L’1_2_ ordered phase is dispersed in the matrix. The ordered phase is more clearly distinguished in a dark-field (DF) TEM micrograph (Fig. 2c) using the (001) reflection (green dotted circle in Fig. 2a). The ordered phase with a diameter of less than 2 nm is finely dispersed in the matrix as shown in the magnified DF image (Fig. 2d). Previous works suggested that the ordered phase can be formed via spinodal decomposition even after quenching^26–28^. In the ordered phase, Al, Fe or Mn, C atoms are located at corner, face-centered and body-centered position of the unit cell, respectively (Fig. 2b). Occupancy of the body centered C atom determines the type of the ordered structure: (1) L1_2_ without C atom; (2) L’1_2_ with partially occupied C atom; (3) E2_1_ with fully occupied C atom. The C atom occupancy in the ordered phase can be experimentally estimated by analyzing the intensity of the superlattice reflections, proportional to square of the structure factor (F_*hkl*_). Since the interstitial C atom in the L’1_2_ phase leads to the difference of the structure factor for the (*hkl*) reflections^29^:

$${{\rm{F}}}_{2n}={|{f}_{{\rm{Al}}}}_{{\rm{Fe}}/{\rm{Mn}}}+{f}_{{\rm{c}}}|\,{\rm{for}}\,(h+k+l)=2n,$$

$${{\rm{F}}}_{2n+1}=|\,{f}_{{\rm{Al}}}-{f}_{{\rm{Fe}}/{\rm{Mn}}}-{f}_{{\rm{c}}}|\,{\rm{for}}\,(h+k+l)=2n+1,$$

the intensity ratio of the {110} to {100} reflections, i.e. I_{110}_/I_{100}_, decreases as the C atom contents increases. Figure 3 shows the estimation of the C atom content in the ordered phase of the present study. The I_{110}_/I_{100}_ of 0.62 is measured from the [001]-zone axis SADP as shown in Fig. 3a. Figure 3b–d show the simulated SADPs of the L’1_2_ phase with decreasing C contents from 20 to 2 at.%, showing that the inverse relation between the I_{110}_/I_{100}_ the C content. Matching between the measured and calculated I_{110}_/I_{100}_ informs the ordered phase contains 1.6 at.% of the body centered C interstitial atoms. Even definite elemental partitioning was not occurred, the carbon ordering took place during quenching due to the fast diffusivity of the C atom^11^. The interstitial C atoms in the ordered phase increase the energy of antiphase boundary (APB) which formed by shearing of the ordered phase by gliding dislocations^12,30,31^. The APB energy dependent upon the atomic configuration of the ordered phase plays an important role in dislocation activities and plasticity of the alloy.

**Figure 4:**
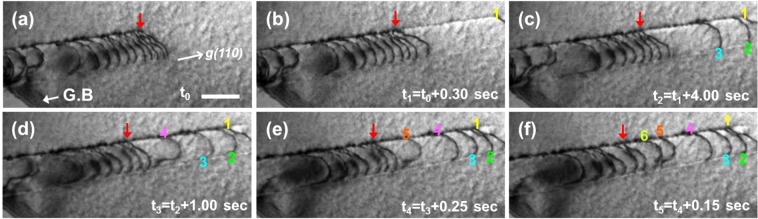
Bright-field (BF) TEM micrographs of the Fe-Mn-Al-C steels at early stage of plastic deformation. (**a**–**f**) Consecutive frames separated by time intervals inserted in each frame. The dislocations are enumerated according to the position in the glide plane. Gliding dislocations are obstructed at a pinning point (red arrow). Length of the scale bar is 200 nm. See the corresponding Supplementary Video 1 for better understanding of the situation.

Figure 4 shows a series of bright-field (BF) TEM micrographs demonstrating early stage of plastic deformation of the Fe-Mn-Al-C steels. Images in Fig. 4 were captured from Supplementary Video 1. Dislocations were generated at the grain boundary and glided on a {111} plane. The crystallographic glide planes with a high dislocation density are generally called as the slip bands (SBs). It is notable that the gliding dislocations were blocked at a pinning point (red arrow). At first, the leading dislocation was suddenly escaped from the pinning point (Fig. 4b). After 4 sec, the second and third dislocations were emitted from the pinning point almost at the same time (Fig. 4c). The forth dislocation was also impeded at the pinning point for 1 sec (Fig. 4d). After the passage of the fourth dislocation, gliding of the following dislocations was not blocked in the vicinity of the pinning point, so the time intervals for the passage of fifth and sixth dislocations through pinning point were decreased to 0.25 sec and 0.15 sec, respectively (Fig. 4e,f). Consequently, the capacity of dislocation pinning was exhausted after passage of four leading dislocations in total. Because any visible obstacle to dislocations motion such as interfaces or dislocations was not found at the pinning point, it is conceivable that the obstruction of the dislocations glides is attributed to the interaction between the dislocations and the nano-sized L’1_2_ ordered phases.

**Figure 5:**
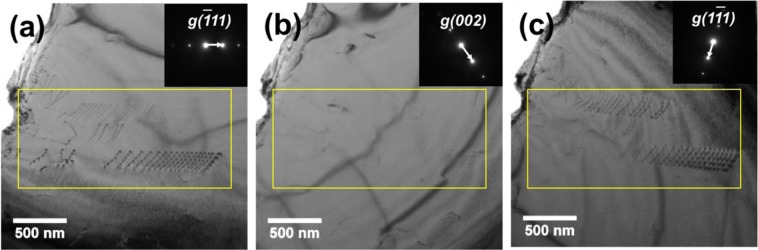
(**a**–**c**) Bright-field (BF) TEM images of the dislocations in slip bands (SADP) with the corresponding two-beam conditioned diffraction pattern (inset image).

Burgers vector (**b**) of the gliding dislocations is determined via *post-mortem* (i.e. τ_ext_ = 0) TEM analysis with the the ***g****·****b*** invisibility criterion. Figure 5a–c are bright-field (BF) TEM images where each of their corresponding two-beam condition is provided as the inset. Dislocations in the slip bands become invisible under $\mathop{{\boldsymbol{g}}}\limits^{{\boldsymbol{\rightharpoonup }}}=(200)$ (Fig. 5b), which gives an evidence that the dislocations are not the Shockley partials. Although the dissociation of perfect dislocations to Shockley partials is energetically more favorable (Frank’s rule)^22^, it is conceivable that the high SFE of the current alloy inhibits the dislocation dissociation.

From the ***g****·****b*** invisibility criterion, the Burgers vector (***b***) of the dislocations is determined as (a/2) <110 >, namely the perfect dislocations in the primary slip system (<110>{111}) of fcc materials. It should be noticed that all of the dislocations have the same contrast under each two-beam condition, meaning that they arrange themselves as dislocation pile-ups^32^. The dislocations in the pile-ups have a mixed character with a large screw component (the angles between the Burgers vector (***b***) and dislocation line vector (***l***) are in the range of 5° to 11°)

**Figure 6:**
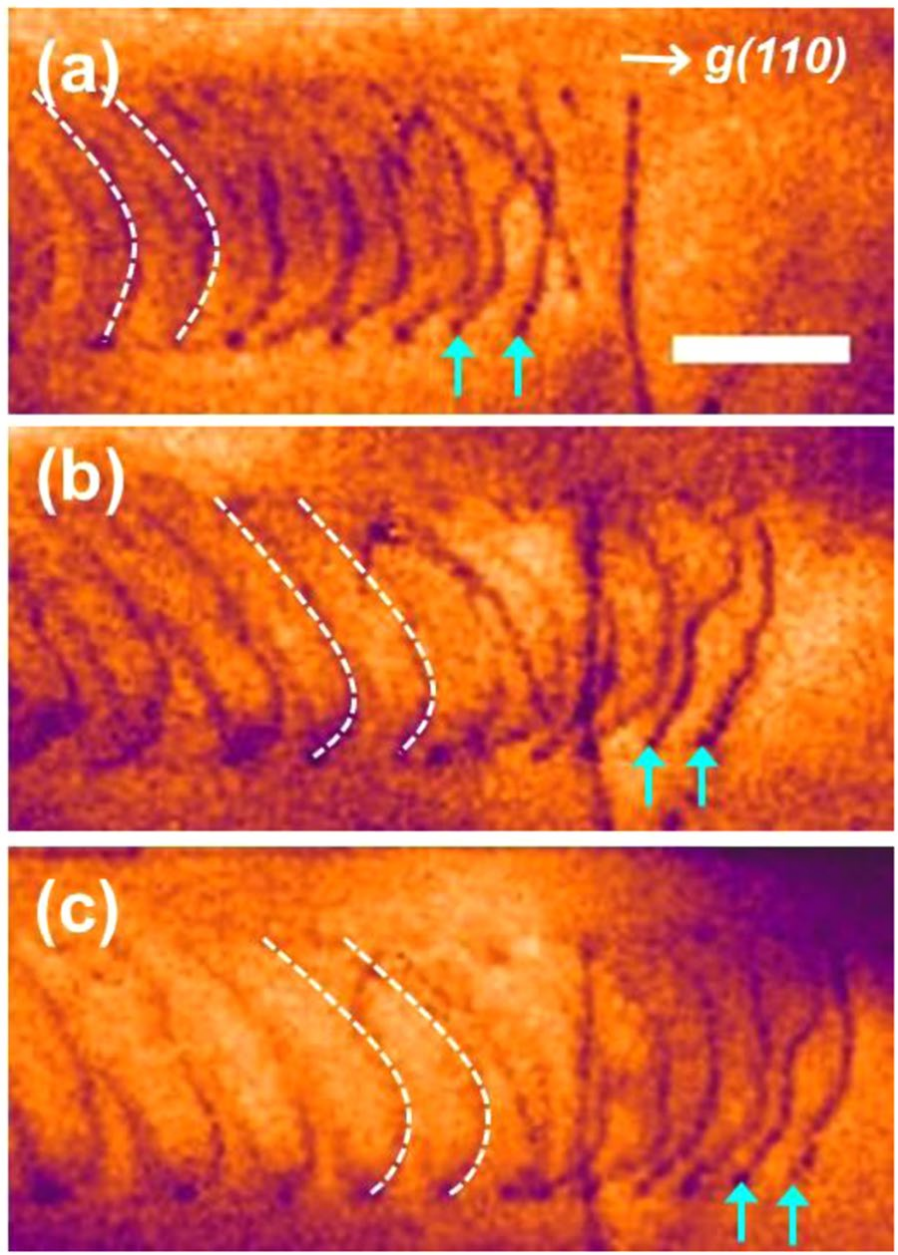
Propagation of the slip band in the form of the planar dislocation glide. (**a**–**c**) Consecutive frames of the propagating slip band front. Curvature of first two dislocations (cyan arrows) is completely different from that of the succeeding dislocations (white dotted lines). Length of the scale bar is 200 nm. See the corresponding Supplementary Video 2 for better understanding of the situation.

Propagation of the slip band in the form of the planar glide is shown in Fig. 6, colored BF TEM images extracted from Supplementary Video 2. The blue-orange icb LookUp Table (LUT) of image processing software (ImageJ) was used to enhance the image contrast and the scene recognition. In this case, velocity of the dislocations gliding was higher than that of the previous example (Fig. 4 and Supplementary Video 1). However, it is remarkable that the curvature of first two dislocations (cyan arrows) was completely different from that of the succeeding dislocations (white dotted lines). Because the curvature of a moving dislocation is determined by equilibrium between the applied shear stress and the dislocation line tension^22^, the deflection of the first two dislocations shows additional resistive stress was exerted on the dislocations. Same as in Fig. 4, any visible obstacle to dislocation glide was not found, meaning that the finely dispersed L’1_2_ ordered phases played a role in the dislocation motion.

Results in Figs 4 and 6 exhibit plastic strains are accommodated by the pronounced planar glide of dislocations in the current Fe-Mn-Al-C alloy. The exclusive dislocation glide on specific planes ({111} slip planes) leaded to formation of the crystallographically aligned slip bands. Instead, transition of the slip planes, i.e. cross-slip, was hardly observed in the course of the slip band propagation. Further, our *in-situ* TEM observation confirmed that the dislocation glide was obstructed by the ordered phases in austenite matrix.

**Figure 7:**
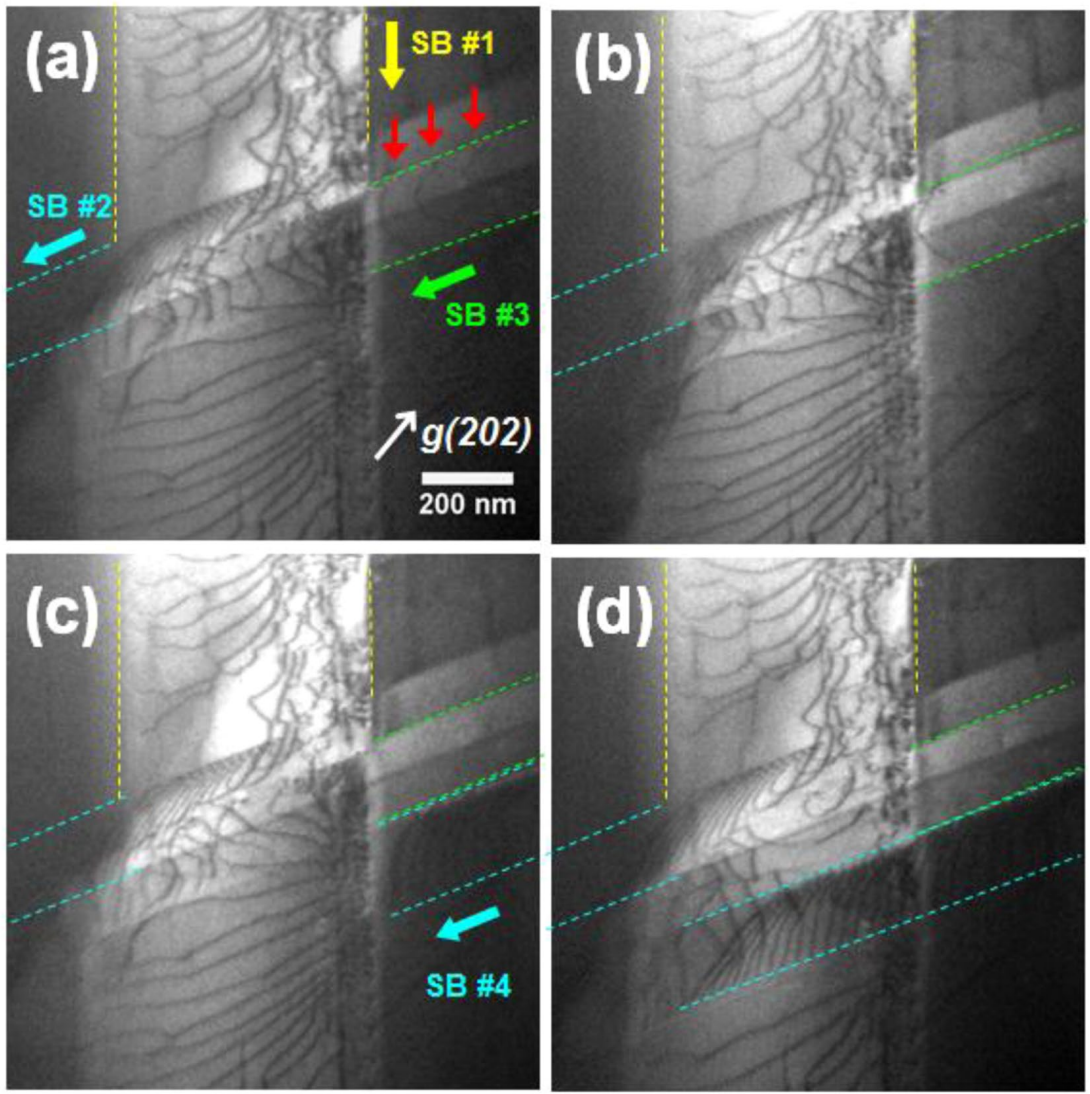
Multiple slip bands formation and their mutual intersection. **(a**–**d)** Consecutive frames of the slip bands intersection. Localized cross-slip of gliding dislocations happen at the slip band intersection. See the corresponding Supplementary Video 3 for better understanding of the situation.

Typical polycrystalline materials accommodate plastic strain by activation of multiple non-coplanar slip systems^3^. In our alloy system, the pronounced planar glides occur on the <110> {111} slip systems. As the dislocations propagating within each slip band (SB), the intersections of the slip bands with different variants necessarily occur in the grain interior. The intersection of the slip bands is visualized in Fig. 7 which was extracted from the Supplementary Video 3. As shown in Fig. 7a, SB #1 (yellow color) and SB #2 (cyan color) had been already formed and intersected each other. As the gliding dislocations in SB #2 were exhausted, dislocations (red arrows) in the newly generated SB #3 (green color) start to glide as shown in Fig. 7a. The gliding dislocations in SB #3 become also exhausted (Fig. 7b) with ongoing deformation, then a new slip band (SB #4) is activated and intersected with the SB #1 (Fig. 7c,d).

**Figure 8:**
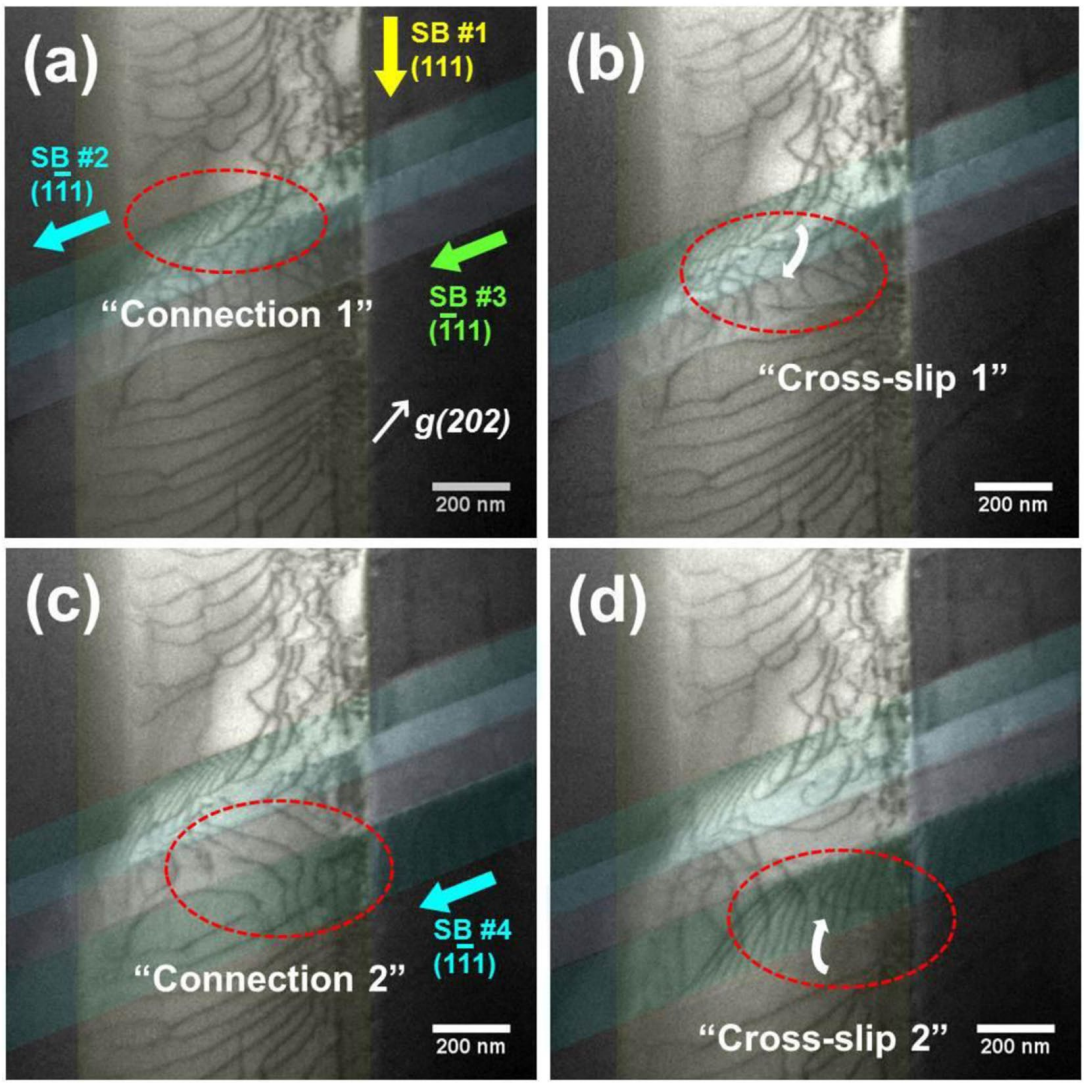
Selected key frames from Supplementary Video 3 showing the dislocations connection and the cross-slip. (**a)** Dislocation connection between the gliding dislocations in the SB #1 and the SB #2. (**b)** Dislocation cross-slip from the SB #1 to the SB #2. **(c)** Dislocation connection between the gliding dislocations in the SB #3 to the SB #4. **(d)** Dislocation cross-slip from the SB #1 to the SB #4.

**Figure 9:**
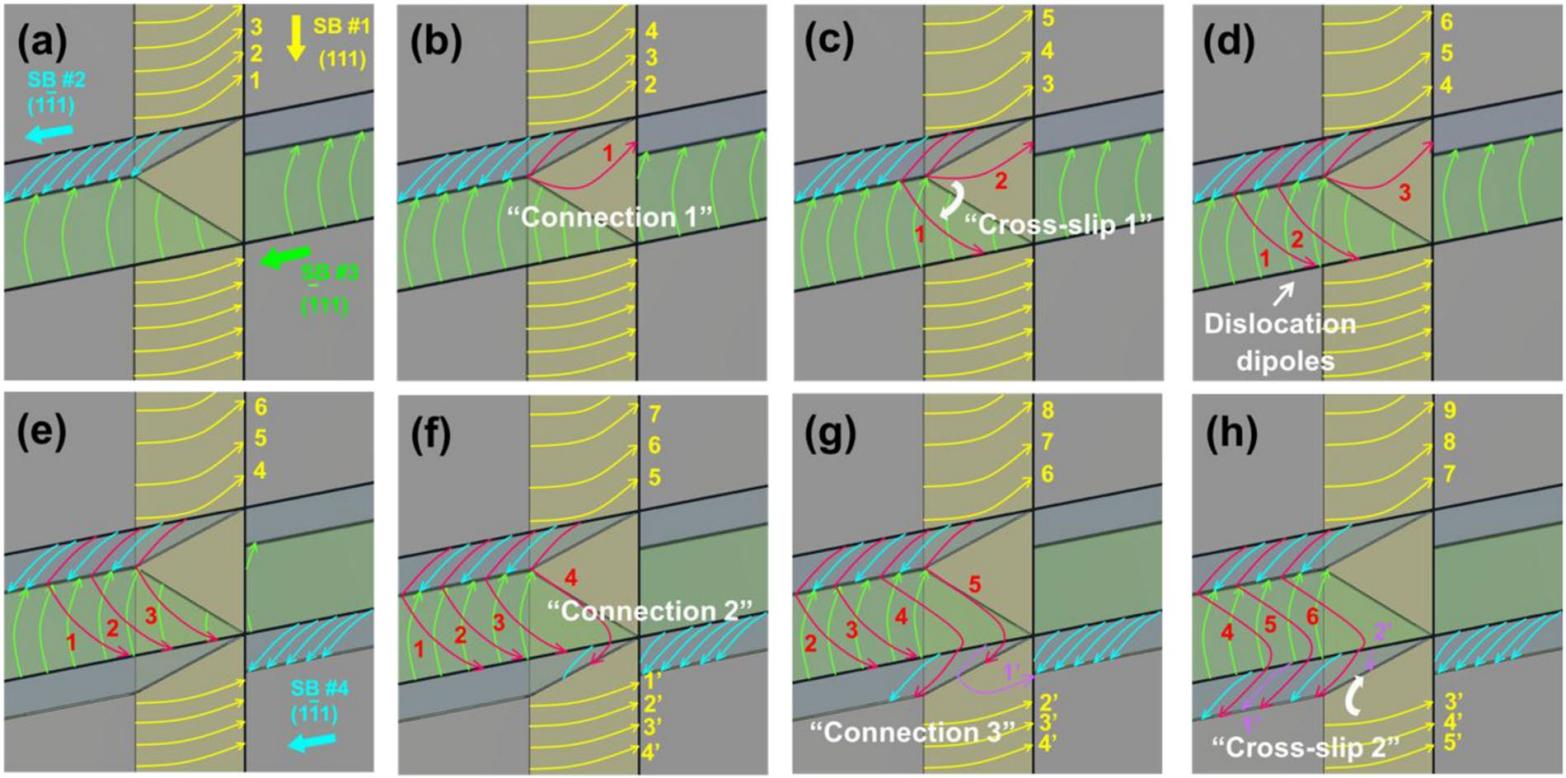
Schematic illustrations of the sequential behaviors of the gliding dislocations at the slip band intersections. **(a)** Initial configuration of dislocations at a slip band intersection. **(b)** Connection between the dislocations in SB #1 and in SB #2. **(c)** Cross-slip of the connected dislocation from SB #1 to SB#3. **(d)** Formation of dislocation multipoles in SB #3 by the consecutive cross-slip. **(e)** Formation of SB #4 by the pronounced planar dislocation glide on $(1\bar{1}1)$ plane. **(f)** Connection between the dislocations in SB #3 and in SB #4. **(g)** Connection between the dislocations in SB #4 and in SB #1 (lower part). **(h)** Cross-slip of the connected dislocation from SB #1 to SB#4. See the corresponding Supplementary Video 3 for better understanding of the situation.

Even the planar glide of slip band has been widely reported^7,10,11^, to the best of our knowledge, in-depth investigations about the dislocation dynamics at the slip band intersection are still scarce. The *in-situ* TEM movie (Supplementary Video 3) contains detailed dislocations behavior at the slip bands intersection. Key frames from Supplementary Video 3 are selected in Fig. 8, showing the stages of dislocations interaction and the process of cross-slip at the slip band intersection. Figure 9 illustrates the sequential steps of the gliding dislocations interaction observed in the movie. Descriptions of the dislocations behavior in the movie are made as follows.

1. Figure 9a shows the initial configuration of slip bands before the dislocation interactions. In this state, three of <110 >{111} slip systems are activated, and the interactions among the slip bands are neglected as a reference state.
2. The gliding dislocations in SB #1 (D-SB #1, yellow arrows) and the dislocations in SB #2 (D-SB #2, cyan arrows) are connected to form the dislocation junction^33–35^ (Figs 8a, 9b**)**. Then, D-SB #1 is pinned by D-SB #2.
3. Then, the pinned D-SB #1 s execute the cross-slip from (111) SB #1 to $(\bar{1}11)$ SB #3 (Figs 8b, 9c), forming the energetically stable dislocation multipole^36,37^ configuration as shown in Fig. 9d. An interesting point is that the cross-slip can be achieved at the slip bands *intersection* although the cross-slip hardly happens during the slip band *propagation*.
4. As deformation proceeds, SB #4 propagates on a $(1\bar{1}1)$ plane which is parallel to the SB #2 (Fig. 9e). The cross-slipped D-SB #1 can be connected again with the gliding dislocations in SB #4 (D-SB #4, cyan arrows in SB #4) as shown in Figs 8c, 9f.
5. Furthermore, D-SB #4 s are connected with the already existing D-SB #1 s which are settled in the low part of the SB #1 (Fig. 9g). It is notable that the activated gliding dislocations (D-SB #4 s) drag stationary dislocations (D-SB #1 s) within a finite distance. After the connection, the D-SB #1 s do cross-slip into the SB #4 (Figs 8d, 9h).

**Figure 10:**
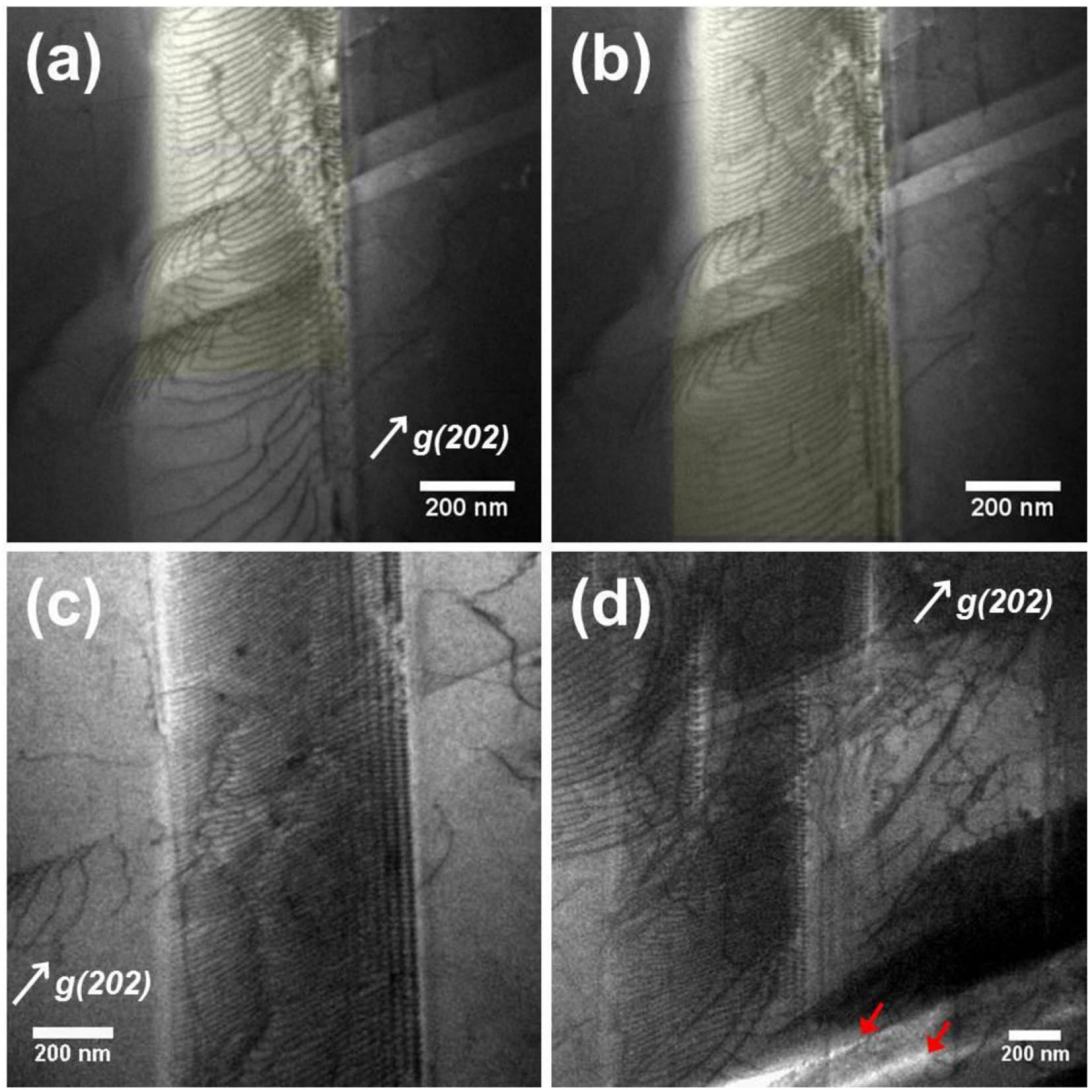
(**a**,**b**) Consecutive frames showing filling-up of a slip band with dislocations. (**c**) Highly dense dislocation wall (HDDW) formed after the dislocation pile-up. (**d**) Multiple HDDWs are settled on the different {111} planes. Cracks propagated along the highly stressed HDDWs (red arrows). See the corresponding Supplementary Video 4 for better understanding of the situation.

As the deformation continued, slip bands were filled with dense pile-up dislocations. As shown in Fig. 10a,b, which was extracted from Supplementary Video 4. A burst of dislocations propagating from the upper-part of the slip band was momently clogged in the vicinity of the slip bands intersection (Fig. 10a). Then, the rest of the slip band was filled again with the dense dislocation array by means of the avalanche-like process (Fig. 10b)^38,39^. As a result, a highly dense dislocation wall (HDDW)^9,19,25,40^ was generated as shown in Fig. 10c. Figure 10d shows the formation of the multiple HDDWs at high stress level. At the high stress level, eventually, cracks initiated and propagated along the highly stressed HDDWs (red arrows).

## Discussions

In general, SFE is known as one of the most important determining factors for dislocation glide mode of the austenitic steels^13–16^. In low or medium SFE materials, dissociations of perfect dislocations into Shockley or Frank partials are preferred, then the planar dislocation glide prevails because the cross-slip of the dissociated Shockley partials is geometrically unfavorable^22^. In high SFE materials, while the dislocation dissociation is prohibited, cross-slip of screw component of dislocations readily occur, leading to the wavy dislocation glide^22^. The SFE of the investigated Fe-Mn-Al-C steels is reported to be about 85 mJ m^−2^ ^8,10,11^, which is higher than that of the high SFE metals such as copper (~60 mJ m^−2^) exhibiting the wavy dislocation glide. If SFE is only concerned, the wavy dislocation glide rather than planar dislocation glide will be expected in the current alloy. However, our *in-situ* TEM observation confirms the pronounced planar dislocation glide could be preferred even in such high SFE material.

In fact, the pronounced planar glide in the Al-containing high Mn steels has been reported in previous investigations^7,8,10,11^. They proposed the “glide plane softening”’ is the main origin of the planar glide^23^ and presented the experimental evidences of the glide plane softening by means of analyzing the paring of dislocations at the slip band front^11,41,42^. Because Burgers vector of the gliding dislocation (a/2 < 110>{111}) is half of the closing vector which maintains perfect lattice symmetry of the ordered phase, shearing of the ordered phase by a gliding dislocation leaves the antiphase boundary (APB). But, when a succeeding dislocation shears the ordered phase again, the original symmetry of the ordered phase can be restored. Therefore, they suggested that the pairs of dislocations, called as superdislocations, should glide together at the slip band front to avoid the formation of the APB^31,43,44^.

However, the dislocations paring was not observed in our *in-situ* TEM experiments; instead it was found that each dislocation moves independently. Yao *et al*.^12^ summarized the effects of the L’1_2_ ordered phase on the coupling strength between the paired dislocations. They reported that the transition from weak to strong coupled regime takes place with ordered phase radius (r) of 6.8 nm, taking *G* (shear modulus) as 70 GPa, *b* (length of the Burgers vector) as 0.26 nm, γ_APB_ (antiphase boundary energy) as 700 mJ/m^2^ (with C atom ordering at the body centered site in L’1_2_ structure). In other words, if the size of the ordered phase is effectively small, i.e. r·γ_APB_ <<*Gb*^2^, the gliding dislocations are weakly coupled even though the γ_APB_ is considerably high. Also, they noticed that the normalized strengthening is proportional to the radius (r) of the ordered phase in the weakly coupled regime. Due to the size of the ordered phase in the current alloy is within the weakly coupled regime (as presented in the session 3.2), the independent movement of dislocations in the slip plane can be rationalized.

In the present study, we can provide direct evidences of the “glide plane softening” phenomena by observing the interaction between the gliding dislocations and the ordered phase. In the pinning case (Fig. 4), an ordered phase (the pinning point) hindered only four leading dislocations, i.e. the precipitate was entirely sheared after passage of the fourth dislocation. After the shearing of the precipitate by leading dislocations, succeeding dislocations did not experience any resistance against their motion, meaning the slip plane had been “softened”. The deflection case (Fig. 6) can also be understood by the glide plane softening. Compared to other gliding dislocations in a slip band, a pair of leading dislocations in the slip band was prominently deflected. This means the two preceding dislocations suffered the resistive stress originated from the fine precipitates at the slip band front, but the succeeding dislocations were free from the hindrance after the shearing of the precipitates. Compared to the pinning precipitate in the Fig. 4, the fine precipitates in Fig. 6 could not effectively block the glide of the dislocations because the resistive strength is proportional to the size of the ordered phase. Also, the stress multiplication effect by the collective dislocations pile-up in the slip band might help to overcome the resistive stress induced by the ordered phase^11,41^. The diameter of the pinning precipitate (Fig. 4) can be estimated to be around 1 nm by counting the pinned dislocations, i.e. 4 (number of pinned dislocations) × 0.26 nm (length of the Burgers vector of the dislocations) = 1.04 nm. Also, the number of the deflected dislocations (Fig. 6) reveals that the diameter of the precipitates in the deflection case is below 0.5 nm i.e. 2 (number of pinned dislocations) × 0.26 nm (length of the Burgers vector of the dislocations) = 0.52 nm. The size estimation of the precipitates coincides well with the TEM results in session 3.2.

Cross-slip of the gliding dislocations at the slip band interactions is another visible evidence of the glide plane softening. As mentioned before, in the present Fe-Mn-Al-C alloys, dislocations are prone to collectively glide on a slip band where the ordered phase clusters were totally sheared by the preceding dislocations. Therefore, cross-slip of individual gliding dislocation in a slip band hardly happens because it needs additional flow stress to shear the ordered phase in the designated slip plane. Nonetheless, the cross-slip might be expected where the easy paths for dislocation glide are connected, i.e. at the slip band intersections. In Figs 8 and 9, we present the cross-slip of dislocation at the slip band intersection, which visualize the planar glide dislocations can do cross-slip where the easy glide paths are connected (i.e. slip band intersection).

Plastic deformation of the investigated Fe-Mn-Al-C alloy can be characterized by the pronounced planar dislocation glide leading to development of the crystallographically aligned slip bands and HDDWs. Because any strain-induced phase transformation or deformation twinning was not observed during deformation, the strain hardening behavior in the present Fe-Mn-Al-C steel can be interpreted from the evolution of dislocation substructure. The classical deformation theory for polycrystalline fcc metals states that the monotonic increase of dislocation density in stage II leads to a constant strain hardening rate, whereas cross-slip and dynamic recovery activated in stage III decrease the strain hardening rate^3,45^. Accordingly, the enhanced strain hardening rate of the current Fe-Mn-Al-C steel can be achieved by further extension of stage II region (or retardation of the transition from stage II to stage III). The pronounced planar glide in the current alloy aids the extension of stage II region with two aspects. At first, dislocations cross-slip is inhibited by the pronounced planar glide^11,46^. In the light of our findings, cross-slip of the gliding dislocations is hardly happened with exception of the cross-slip at the slip band intersections. Consequently, frequency of dislocation annihilations by cross-slip is strongly reduced, delaying the dynamic recovery. Second, extensive multiple slip band formation by the planar dislocation glide suppresses strain localization during deformation, creating evenly distributed dislocations within grains. Especially, the localized cross-slip of gliding dislocations at the slip band intersections, which is figured out by the present study, enables the slip bands to propagate in the grains without blocking each other.

Furthermore, it is worthwhile to discuss about the type of interactions between the gliding dislocations. There have been controversies about whether the passing stress (long-range interaction) or the cutting stress (short-range interaction) mainly contributes to the strain hardening of the dislocation-mediated materials^11,21,47–51^. From the results of our *in-situ* TEM experiments, it is shown that mutual shearing between dislocations or formation of Lomer-Cottrel locks^52^ is hardly happened during the deformation. Also, it was confirmed that the plastic deformation was realized by the gliding of dislocations pile-ups (i.e., the Burgers vector of the dislocations is identical). Contrary to low energy dislocation structures (LEDS) such as Taylor lattice, dislocation pile-ups produce large and long-range stress field^22,32^. Therefore, our *in-situ* TEM experiment supports the earlier study^11^ in which the long-range stresses from the stored slip bands are the main source for strain hardening in the Fe-Mn-Al-C steels.

## Conclusions

In the present study, we investigated the microstructure evolution of a Fe-Mn-Al-C steel (Fe-32Mn-8.9Al-0.78 C (wt.%)) during plastic deformation by means of TEM analysis including *in-situ* straining TEM. Following conclusions can be drawn.

- The investigated steel exhibits outstanding mechanical properties including high strength and high ductility (yield strength of 425 MPa, maximum strength of 1319 MPa and total elongation of 49%). Especially, strain hardening rate reaches to the maximum value of 2000 MPa.
- It is confirmed by the *in-situ* straining TEM analysis that the plastic deformation is accommodated by the pronounced planar dislocation glide followed by the formation of slip bands and HDDWs. Neither martensitic transformations nor deformation twins were observed during plastic deformation. The localized cross-slip of dislocations at the slip band intersection is observed, revealing that the slip bands can propagate in a grain without blocking each other.
- Experimental evidences of the glide plane softening are provided. Pinning and deflection of gliding dislocations at the slip band front visualize the interaction between dislocations and the nano-sized ordered phase precipitates. Also, the activation of cross-slip at the slip band intersections indicates that dislocations can transfer the slip plane when the slip planes are softened by the shearing of the precipitates by the leading dislocations.
- The enhanced strain hardening behavior of the current alloy is attributed to the pronounced planar glide which extends the deformation stage II region. That is, multiple slip band formation by the planar dislocation glide delays premature failure and dynamic recovery by suppressing strain localization. The present study shows that the long-range stress field from the dislocation pile-ups in the slip bands rather than the cutting stress from the dislocation interactions such as Lomer-Cottrel locks is considered to be the main contribution to the strain hardening of the austenitic Fe-Mn-Al-C steels.

## Materials and Methods

The investigated alloy whose chemical composition of Fe-32Mn-8.9Al-0.78 C (wt.%) was melted in an induction furnace and cast into 60 kg ingot (180 mm dia. × 250 mm height) under vacuum. The ingot was re-heated for 5 h at 1220 °C and forged into a block with dimensions of 70 × 120 × 750 mm. Plate-shaped samples (70 × 120 × 20 mm) were cut from the forged sample and homogenized at 1050 °C for 5 h and subsequently water quenched. Cylindrical tensile samples were prepared with gauge dimension of 6.25 mm in diameter and 25 mm in length. Tensile tests were performed at room temperature with a strain rate of 0.008 s^−1^ using a tensile test machine (INSTRON 5982, Canton, MA). The microstructures of the as-quenched samples were investigated using TEM. For a conventional *post-mortem* TEM analysis, discs with a diameter of 3 mm were mechanically polished to about 100 μm thickness and electrochemically etched by a twin-jet electrolytic polishing machine (TenuPol-5, Struers). The electrochemical etching was conducted at 10 V and 70 mA with a mixed solution of 10% perchloric acid and 90% methanol at −20 °C. Samples for the *in-situ* TEM experiments with specific dimensions (3 mm × 12 mm) were prepared by punching the polished foil using a custom-made foil puncher. The *in-situ* TEM samples were finally etched to make electron transparent regions with aforementioned etching condition. A TEM (JEM-2100, JEOL Ltd.) was used at acceleration voltage of 200 kV and an *in-situ* straining TEM stage (straining *in-situ* holder- model 654, Gatan, Inc.) was used for the *in-situ* experiments. Selected area diffraction pattern (SADP) simulations were conducted using SingleCrystal (CrystalMaker Software Ltd.).

## Supplementary information

Supplementary Video1

Supplementary Video2

Supplementary Video3

Supplementary Video4
